# Validation of a new method for the detection of Ethyl glucuronide in larvae of *Lucilia sericata* as a marker of ante-mortem alcohol consumption

**DOI:** 10.1016/j.heliyon.2023.e20802

**Published:** 2023-10-07

**Authors:** Alice Cerioni, Erika Buratti, Gianmario Mietti, Marta Cippitelli, Mariano Cingolani, Rino Froldi, Roberto Scendoni

**Affiliations:** Forensic Medicine Laboratory, Institute of Legal Medicine, University of Macerata, Via Don Minzoni 9, 62100 Macerata, Italy

**Keywords:** Entomotoxicology, Ethanol, Forensic toxicology, Ethyl glucuronide, Ultra-high-performance liquid cromatography, Larvae

## Abstract

Larvae and insects are an important and alternative biological matrix in the development anpost-mortem forensic toxicology. They are very useful when conventional matrices are not available, in particular when the loss of biological fluids, due to the decomposition of corpses or to a traumatic death, occurs. The purpose of this study is to develop and validate an analytical method in Ultra High-Performance Liquid Chromatography at High Resolution Mass Spectrometry (HPLC/HR-MS) for the research and quantification of Ethyl glucuronide (EtG) on larvae. The criteria taken into consideration for the validation are linearity, quantitation limits (LOD and LLOQ), accuracy, precision, carryover, interferences and ionization suppression/enhancement. The method was shown to be linear within the tested range, with a coefficient of determination higher than 0.99. LOD was 2 pg mg^−1^, while LLOQ was 5 pg mg^-^1. Accuracy, precision and ionization/suppression enhancement fulfilled the criteria indicated in the guidelines used for the validation. The establishment and validation of this method allowed the identification of Ethyl glucuronide on the larvae of *Lucilia sericata* (Calliphoridae) of a subject found death in an advanced state of decomposition.

## Graphical abstract

**Figure:**
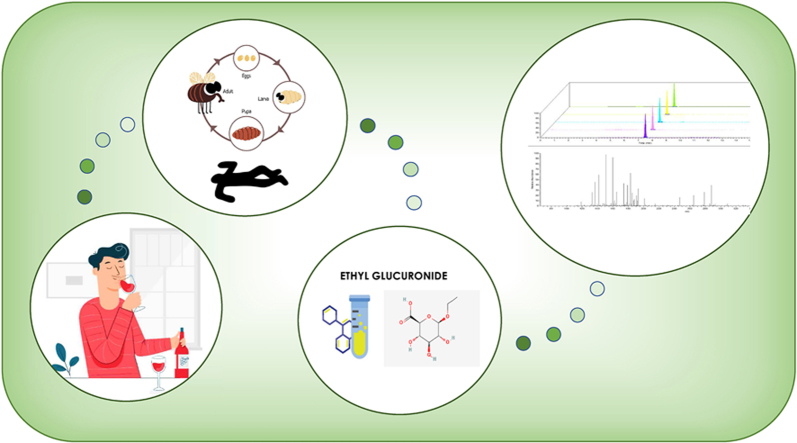

## 1 Introduction

Ethanol is the most frequently used psychoactive substance [1] and represents a serious and major issue in forensic medicine. Alcohol use and abuse is directly or indirectly correlated with a lot of forensic cases and play a crucial role in suicides, violent crimes, trauma related deaths and drowning [2]. Therefore, the evaluation of ethanol in biological samples and the interpretation of the results have a fundamental role in forensic toxicology. This is sometimes particularly difficult and several factors must be taken into account, including the condition of the body and the time elapsed between the death and the autopsy [3].

Entomotoxicology is a section of entomology that studies the application of toxicological analysis to cadaveric insects, in order to identify drugs and toxins present in tissues [4]. If the cadaver is in a particularly advanced stage of decomposition or in traumatic deaths, biological fluids and solid organs may no longer be available for traditional toxicological analysis. In these cases, insect specimens can be used for different types of toxicological analysis, mainly for qualitative analysis, as no correlation has yet been found between the concentration of the drug in the larvae and the amount in the tissue where the larvae were feeding [5]. In case of skeletonized or highly decomposed subjects, the analysis of insects and larvae allows to have less interference and a reduced matrix effect than the putrefying biological cadaveric samples during the analysis [6].

In addition, different species of bacteria, as a result of the liquefaction of tissues and the consequent invasion of various body tissues from the intestine compartment, can produce ethanol [7]. To discriminate post-mortem ethanol production from ante-mortem alcohol intake, data on ethanol and various alcohol abuse markers obtained from different biological matrices are generally compared [8]. However, in case of highly decomposed bodies, the main biological matrices are generally not available and the larvae may represent a valid alternative matrix. Very few studies are present in literature on the detection of markers of alcohol abuse on larvae. The purpose of this work is to set up and validate a simple method to detect the presence of one of the main markers of alcohol consumption, Ethyl glucuronide (EtG), on necrophagous insects. EtG is a direct non-oxidative ethanol metabolite, which is formed by the combination of ethanol with glucuronic acid with the mediation of UDP-glucuronyl transferase (UGT) [9]. Numerous studies have shown that the determination of Ethyl glucuronide in cadaveric biological fluids indicates an ante-mortem intake of alcohol [10,11]. Ethyl glucuronide research on larvae might therefore be useful for the same purpose when biological fluids are not available.

## 2 Materials and methods

### 2.1 Materials

#### 2.1.1 Calibration, quality control and real samples

Matrix-matched calibration (CAL) and quality control (QC) samples were freshly prepared by fortifying 100 mg of blank larvae of *Lucilia Sericata* (Calliphoridae) with different concentrations of an Ethyl glucuronide solution (0.1 ng μL^−1^). Details on calibration and quality control samples are shown in Table 1.

**Table 1:** Concentrations of the calibration and quality control sample.

	Ethyl glucuronide (pg mg^−1^)
CAL1	5
CAL2	30
CAL3	100
CAL4	500
CAL5	1000
CAL6	1500
QClow	5
QCmedium	100
QChigh	1000

Analysis were performed on *Lucilia sericata* larvae recovered from the putrefied body of a 44-year-old man found dead in July 2022. On behalf of the judicial authority, our laboratory was requested to carry out toxicological tests on the biological samples taken from the corpse. The larvae were taken from the eye sockets of the corpse. The larvae collected were immediately frozen. The man was found positive for ethanol on blood and for Ethyl glucuronide on hair matrix. Following toxicological analysis and autopsy, acute alcohol poisoning was established as the cause of death.

#### 2.1.2 Chemicals and reagents for UHPLC analysis

Ethyl glucuronide and its internal standard, Ethyl glucuronide-d5 (EtG-d5), were acquired from Sigma Aldrich (Italy). Methanol for analysis, water for analysis, methanol for HPLC, ultrapure water for HPLC, acetonitrile for HPLC and formic acid were obtained from Carlo Erba (Italy). All reagents were of analytical grade and stored according to the manufacturer's instructions.

### 2.2 Methods

#### 2.2.1 Sample preparation and extraction

For larvae analysis, 200 mg of sample were firstly washed with water and with methanol. After evaporating all the methanol, the samples were mechanically homogenized. Then they were extracted by adding 600 μL of ultrapure water for HPLC 80 μL of ultrapure methanol for HPLC and 20 μL of internal standard (EtG-d5) in aqueous solution (1 ng μL^−1^). The samples were incubated at room temperature overnight, then sonicated for 1 h and centrifuged at 13,000 rpm for 10 min. After centrifugation, 500 μL of the supernatant were taken, placed in a 1.5 mL barbed vial and dried at 55 °C using the evaporator Savant SPD121P of Thermo Scientific. Finally, the samples were resuspended with 50 μL of water with 0.1 % (v/v) of formic acid (Phase A) for injection into UHPLC-HRMS.

#### 2.2.2 Analysis by UHPLC-HRMS

The Thermo Scientific Dionex Ultimate 300 (UHPLC) chromatographic system was used for the analysis of the samples, coupled with the Thermo Exactive Plus Orbitrap (HR-MS) analyzer using the Luna Omega 3 μm Polar C18 (50 × 2.1 mm) chromatographic column by Phenomenex. Phase A consists of H_2_O + 0.1% (v/v) formic acid, while phase B consists of acetonitrile + 0.1% (v/v) formic acid. The column temperature was set at 25 °C and the flow at 0,4 mL min^−1^. The elution gradient is shown in Table 2.

**Table 2:** UHPLC elution gradient.

TIME	PHASE A (%)	PHASE B (%)
0–1	99	1
1–4	5	95
4–6	5	95
6–7	99	1
7–10	99	1

For the identification of EtG and its internal standard (EtG-d5) the exact mass (EM) and the masses of the ions produced (PI) by in source collission induced dissociation (50 eV) with a tolerance of 5 ppm, were used. The values taken into account for analytes are: 221.06668 (EM), 75.00877, 85.02950, 113.02442 (PI) for Ethyl glucuronide and 226.09805 (EM) for Ethyl glucuronide deuterate.

### 2.3 Validation

Method validation experiments were conducted as reported by the “Standard Practices for Method Validation in Forensic Toxicology” published by the AAFS Standards Board (ABS) [12]. The method was validated for linearity, quantitation limits (limit of detection (LOD) and lower limit of quantitation (LLOQ)), accuracy, precision, carryover, interferences and ionization suppression/enhancement.

#### 2.3.1 Calibration model

The homogeneity of variances was investigated with Cochran test. Since homoscedasticity has been demonstrated, a simple linear regression model using the least squares has been applied as calibration model. The calibration curve for Ethyl glucuronide was made using 100 mg of blank matrix-matched calibrator samples fortified with six different non-zero concentrations ranging from 5 pg mg^−1^ to 1500 pg mg^−1^. Five replicates at each concentration were analysed.

#### 2.3.2 Limits

Sensitivity was assessed by determining LOD and LLOQ. The LOD, which is the minimum measured concentration from which the presence of the analyte can be deduced with reasonable statistical certainty, was calculated fortifying samples with decreasing concentrations of EtG and identifying the smallest concentration that produce a positive result. The LLOQ was defined as the value of the lowest non-zero point of the calibration curve.

#### 2.3.3 Bias and precision

Accuracy and precision studies were performed analysing spiked matrix samples at three diverse concentrations (QClow, QCmedium and QChigh) in triplicates over five different days. The highest acceptable value for bias is ±20 % at each concentration. Accuracy is expressed as bias, which is the systematic deviation from the true value. Precision is indicated as the coefficient of variation (%CV). The %CV shall be less than 20 % at each concentration. In details, two types of precision studies were carried out, intra-day and inter-day precision.

#### 2.3.4 Carryover

Carryover studies were assessed using blank matrix samples analysed immediately after the injection of high concentration samples.

#### 2.3.5 Interference studies

To evaluate matrix interferences ten blank matrix samples were analysed to prove that there were not any interferences from the matrix. In order to study the interferences from stable-isotope internal standards, a single blank matrix sample fortified with EtG-d5 was analysed and observed if there was a signal of EtG. To evaluate interferences from other analytes used in the laboratory, fortified blank matrix samples with Ethyl sulfate, Ethyl miristate, Ethyl palmitate, Ethyl oleate and Ethyl stereate were analysed.

#### 2.3.6 Ionization suppression/enhancement

Ionization suppression/enhancement studies were performed in order to evaluate the presence of co-eluting compounds. A post-extraction addition approach was chosen, using two sets of samples prepared at two different concentrations (QClow and QChigh). The first set is made from spiking analyte in mobile phase, injected six time each. Set two consisted of ten blank matrix samples, which were extracted and then spiked with Ethyl glucuronide and the internal standard, injected once each. Average peak areas were used to calculate the percentage of ionization suppression/enhancement for each concentration.

## 3 Results and discussion

### 3.1 Calibration model

A calibration curve was constructed with a range of linearity between 5 pg mg^−1^ and 1500 pg mg^−1^ and the relative regression equation was calculated. The coefficient of determination (R^2^) was higher than 0.99, precisely 0.9996, thus allowing a reliable quantification. The method was shown to be linear within the tested range.

### 3.2 Limits

**Fig. 1:**
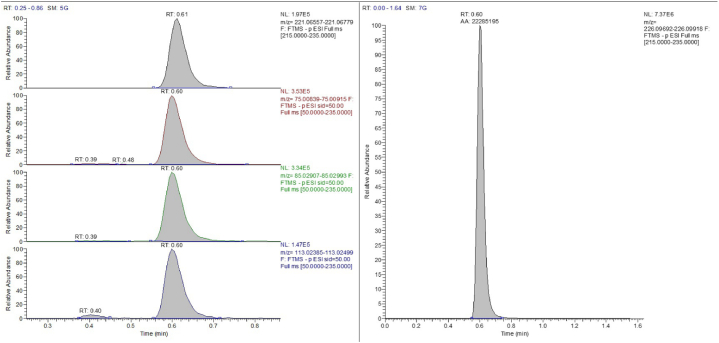
Chromatogram of a blank sample fortified with Ethyl glucuronide at the concentration of the first point of the calibration curve (LLOQ).

To define the LOD, three different blank matrix sources were fortified with decreasing amounts of EtG and analysed over three runs. LLOQ studies were carried out fortifying three different blank matrix samples with EtG at the concentration of the first point of the calibration curve and analysing them over three runs (Fig. 1). All detection, identification, bias and precision criteria were met. LOD was 2 pg mg^−1^, while LLOQ was 5 pg mg^−1^.

### 3.3 Bias and precision

Bias was determined for each concentration and was lower than 20 %. Precision was found not higher than 20 % at each concentration. Precision studies were assessed both in the same analytical session (intra-day precision) and between different analytical sessions (inter-day precision). They were determined through the one-way ANOVA approach.

### 3.4 Carryover

Carryover studies were performed analysing a potential contamination in the blank samples. In details, carryover was evaluated for the concentration of 500 pg mg^−1^, 1000 pg mg^−1^ and 1500 pg mg^−1^. No carryover was detected.

### 3.5 Interference studies

**Fig. 2:**
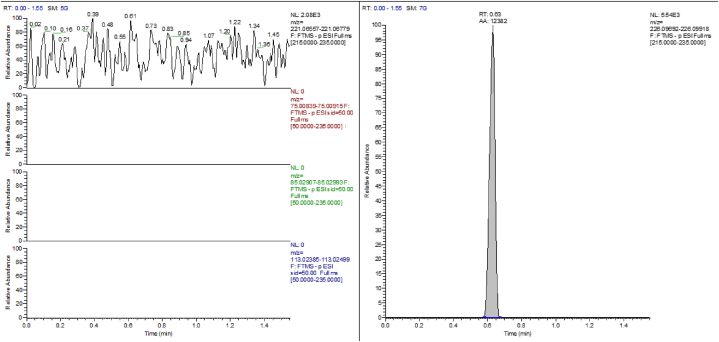
Interference studies from stable-isotope internal standards. The figure shows the chromatogram of a blank sample fortified only with EtG-d5.

For the evaluation of matrix interferences, no interfering signals were noted in the blank matrix samples. As the method employs EtG-d5 as internal standard, also interference studies from stable-isotope internal standards were established. No interferences were observed (Fig. 2). To evaluate the interferences from other substances commonly used in the laboratory matrix blank samples fortified with Ethyl sulfate, Ethyl miristate, Ethyl palmitate, Ethyl oleate and Ethyl stereate. These compounds show different retention times than EtG, so no interferences were observed.

### 3.6 Ionization suppression/enhancement

The two sets of samples described above for the assessment of suppression/enhancement experiments were injected to note the average peak areas of EtG. The percentage of suppression/enhancement was calculated for the low and high concentration, resulting in an enhancement of 6.4 % for the low concentration and an enhancement of 4.4 % for the high concentration. The positive results suggested the presence of some enhancement, which did not exceed ±25 % and so it is meaningless.

All the validation parameters are shown in Table 3.

**Table 3:** Validation parameters of the method.

Validation parameters	EtG
Linearity range	5–1500 pg mg^−1^
Limit of detection	2 pg mg^−1^
Limit of quantification	5 pg mg^−1^
Accuracy low concentration	11.1%
Accuracy middle concentration	−12.8%
Accuracy high concentration	−16.7%
Intra-day precision low concentration	3.3%
Inter-day precision low concentration	5.1%
Intra-day precision middle concentration	1.5%
Inter-day precision middle concentration	1.2%
Intra-day precision high concentration	2.1%
Inter-day precision high concentration	2.6%
Ionization suppression/enhancement (low concentration)	6.4%
Ionization suppression/enhancement (high concentration)	4.4%

### 3.7 Proof of applicability

**Fig. 3:**
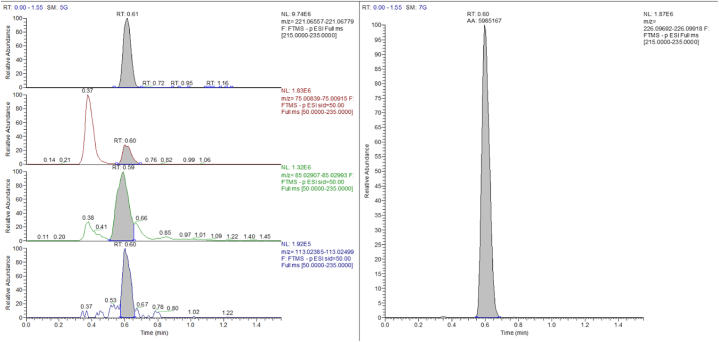
Chromatogram of the real sample.

To verify the applicability of the method, larvae found on the body of a deceased subject, known to be an alcoholic subject, were analysed. The man had a blood alcohol concentration of 3,23 g L^−1^ and was found positive for EtG in hair (with a concentration of 698 pg mg^−1^), one of the few other matrices available. For these reasons he was found to be drunk at the time of death and he has been considered a chronic alcohol abuser. The results of the application of the method to the larvae of this cadaver showed the presence of Ethyl glucuronide on this matrix. The measured concentration is 524 pg mg^−1^ (Fig. 3). This is one of the very first works that has found the presence of Ethyl glucuronide on a real case, that is on the larvae taken from the body of a deceased subject. Ethyl glucuronide is a very reliable marker for monitoring chronic alcohol abuse, characterized by high sensitivity and specificity. It has been found to be of fundamental importance as a marker of ante-mortem alcohol intake [13]. This marker allows to differentiate an in vivo consumption of ethanol from a post-mortem alcohol formation, which can be caused by putrefactive phenomena due to a series of microorganisms. After death it is possible that bacteria from the gut reach the adjacent tissues and the blood circulation, resulting in a production of ethanol via microbial contamination and fermentation [3]. So, in case of corpses in an advanced state of decomposition, where the most common biological matrices (blood, urine and vitreous humor) are not always available, EtG analysis on the larvae might be an alternative for the diagnosis of ante-mortem alcohol ingestion. Ethyl glucuronide is particularly stable in the various biological matrices, if samples are stored properly in the fridge [14,15]. In addition, Ethyl glucuronide does not appear to form post-mortem during putrefactive phenomena or during the storage of samples in the laboratory [10]. In particular, the use of EtG on larvae could be very useful and informative when low concentrations of ethanol are found in the blood, so in cases where it could have formed post-mortem. EtG could be of great utility especially in cases of zero tolerance policies, where it is of fundamental importance to verify that the ethanol found in the blood has been actually assumed by the subject and not produced post-portem by bacteria during putrefactive phenomena.

However, due to the lack in literature of studies concerning the determination of Ethyl glucuronide on larvae, attention should be paid to the interpretation of the resulting data. Nothing can be said about the state of acute alcohol intoxication at the time of death following the finding of a positive EtG on the larvae. In fact, no correlation is known between the amount of Ethyl glucuronide detected in the larvae and the amount of Ethyl glucuronide present in other biological matrices of the same subject, as well as the amount of alcohol taken by the subject. It is necessary to carry out further studies to deepen knowledge about the metabolic processes of ethanol by the larvae. In addition, it will be necessary to examine the interpretation of the quantitative data of Ethyl glucuronide in larvae in relation to the facts of legal medical interest on the use and/or abuse of alcohol.

## 4 Conclusions

Forensic entomology is assuming an increasingly relevant role in post-mortem toxicology, especially when the decomposition processes in the cadaver have made the conventional specimens used for toxicological analysis unavailable [16]. The collection and use of insects is strongly recommended as an alternative matrix for the qualitative analysis of drugs [17]. A great advantage in the employment of this alternative matrix is that it is generally present in large quantities and remains for a long time.

In this work, the application of the validated method allowed to detect the presence of EtG on the larvae of a deceased subject. Surely further studies are needed to apply the method to a larger number of real cases and to deepen the issue about the correlation of the quantitation of Ethyl glucuronide found on the larvae and the state of chronic alcohol abuse.

## Data availability statement

The data underlying this article will be shared on reasonable request to the corresponding author.

## Funding

This research did not receive any specific grant from funding agencies in the public, commercial, or not-for-profit sectors.

## Declaration of competing interest

The authors declare that they have no known competing financial interests or personal relationships that could have appeared to influence the work reported in this paper.
